# Congenital toxoplasmosis among Iranian neonates: a systematic review and meta-analysis

**DOI:** 10.4178/epih.e2019021

**Published:** 2019-05-17

**Authors:** Shahabeddin Sarvi, Tooran Nayeri Chegeni, Mehdi Sharif, Mahbobeh Montazeri, Seyed Abdollah Hosseini, Afsaneh Amouei, Zahra Hosseininejad, Davood Anvari, Reza Saberi, Shaban Gohardehi, Ahmad Daryani

**Affiliations:** 1 Toxoplasmosis Research Center, Mazandaran University of Medical Sciences, Sari, Iran; 2 Department of Medical Parasitology, School of Medicine, Mazandaran University of Medical Sciences, Sari, Iran; 3 Student Research Committee, Mazandaran University of Medical Sciences, Sari, Iran; 4 Department of Parasitology, School of Medicine, Sari Branch, Islamic Azad University, Sari, Iran

**Keywords:** Toxoplasmosis, Incidence, Neonate, Systematic review, Iran

## Abstract

Toxoplasmosis is a serious zoonotic disease that can lead to abortion and congenital disorders and has a widespread global distribution in humans and animals. The objective of this review was to investigate the incidence of toxoplasmosis in Iranian neonates in order to obtain a comprehensive assessment of the overall situation of the disease for use in developing future interventions. Original studies investigating the incidence of *Toxoplasma gondii* infections in Iranian neonates were systematically searched in a number of English-language and Persian-language electronic databases. The search process resulted in the inclusion of a total of 11 studies in the systematic review, 10 of which were entered into the meta-analysis. The reviewed articles included 2,230 Iranian neonates investigated through January 1, 2018. Based on the retrieved studies, the overall weighted incidence rates of toxoplasmosis in the Iranian neonatal population and neonates with suspected congenital toxoplasmosis were estimated to be 0.64% (95% confidence interval [CI], 0.31 to 1.09) and 4.10% (95% CI, 2.68 to 5.77), respectively, using a fixed-effects model. The findings of the reviewed studies demonstrate that the incidence of toxoplasmosis is high in Iranian neonates. Accordingly, it can be concluded that toxoplasmosis is a serious public health concern that has been ignored by the Ministry of Health. Therefore, it is essential to perform further studies, in addition to implementing screening and detection programs, using standardized methods to estimate the incidence of toxoplasmosis in Iran and to determine its associated risk factors.

## INTRODUCTION

Toxoplasmosis is a global endemic disease that infects roughly one-third of the total world population according to seroepidemiological studies [1,2]. Intermediate hosts can acquire the infection by eating raw or weakly cooked meat contaminated with oocysts released from the feces of infected definitive hosts or by consuming food and drink saturated by *Toxoplasma gondii* (*T. gondii*) cysts [3,4]. Other transmission routes include congenital transmission of the disease from mother to fetus during pregnancy, organ transplantation, and infected blood [4].

Congenital toxoplasmosis (CT) is defined as the infection of a fetus, newborn, or infant aged under 1 year with *T. gondii*. The detection of *T. gondii* in bodily tissues or fluids can be accomplished by several methods, including polymerase chain reaction (PCR), inoculation in mice, cell culture, and immunocytochemistry. In addition, the observation of specific immunoglobulin M (IgM) or immunoglobulin A (IgA) antibodies, specific immunoglobulin G (IgG) antibodies, and persistent IgG positivity until 1 year of age is indicative of CT [5].

CT is caused by maternal infection during gestation. The prevalence of *T. gondii* infections in pregnant women varies from 0.79% to 84% across different regions in the world [6,7]. This infection also has different incidence rates in various countries; for instance, 2.90, 5.50, and 0.73 neonates per 10,000 live births in France, Poland, and Sweden, respectively, are born infected with this disease [5,8,9].

Factors affecting the transmission of the infection from mother to fetus include the time of maternal infection during gestation, maternal immunological status, the age of the embryo at the time of transmission, and number and virulence of parasites transmitted to the embryo [10]. When *T. gondii* infection occurs during the first and second trimesters of pregnancy, it is accompanied by severe manifestations, such as low birth weight, hydrocephaly, intracranial calcifications, and retinochoroiditis, which are recognizable at birth [6]. In contrast, neonates infected in the third trimester of pregnancy do not show symptoms of the disease upon birth. Instead, they develop intracranial calcifications, hearing impairments, developmental delays, and visual disorders later in life [11,12]. Furthermore, CT can result in abortion, fetal death, and abnormalities (e.g., blindness and severe cognitive impairment) occurring after birth [4,13]. The definitive diagnosis of CT in infants can be accomplished through a PCR assay (of peripheral blood, cerebrospinal fluid, and urine), along with serological tests [14].

The seroprevalence rates of *T. gondii* infection have been reported to be 39.3% and 41.0% in the Iranian general population and pregnant women, respectively [15,16]. Accurate estimations of the seroprevalence rate of toxoplasmosis in various populations could help physicians diagnose, manage, and control this infection and its sequelae [16]. With this background in mind, the present review was conducted to achieve 2 goals: (1) to evaluate the incidence of *T. gondii* among infants with suspected intrauterine infections (<1 year), neonates born with major congenital malformations, and neonates born of suspected or infected mothers with *T. gondii* infection; and (2) to determine the incidence of toxoplasmosis in infants born to healthy mothers referred to the hospital for childbirth.

## MATERIALS AND METHODS

### Search strategy

The PRISMA (Preferred Reporting Items for Systematic Reviews and Meta-Analyses) guidelines were used to conduct this study [17] (Supplementary Material 1). Our search was limited to articles written in the Persian and English languages. Therefore, publications investigating the prevalence of *T. gondii* infections among neonates in Iran through January 1, 2018, were searched in English-language databases, including PubMed, ScienceDirect, Springer, and Google Scholar, as well as in Persian-language databases, including Magiran, Scientific Information Database (SID), and Iranian Research Institute for Scientific Information and Documentation (IranDoc). The search process was carried out using the following keywords: “toxoplasmosis,” “*Toxoplasma gondii*,” “*T. gondii*,” “congenital toxoplasmosis,” “newborn,” “neonate,” “infant,” “fetus,” “meta-analysis,” “systematic review,” “Iran,” and “Islamic Republic of Iran.”

### Inclusion and exclusion criteria

Following the removal of duplicate entries, original studies and brief reports were evaluated according to the following inclusion criteria: (1) investigation of the incidence of *T. gondii* in Iranian neonates; (2) assessment of only mothers and their infants; (3) diagnosis of toxoplasmosis by performing PCR on amniotic fluid or detecting IgG and/or IgM antibodies against *T. gondii* in the serum, and cord blood; and (4) adoption of a cross-sectional design. The exclusion criteria were: (1) irrelevancy; (2) publication in languages other than English or Persian; (3) inclusion of aborted fetuses; (4) lack of suitable data; and (5) review articles.

### Study selection and data extraction

After the retrieval of the articles meeting the inclusion criteria, their titles and abstracts were independently and carefully examined for eligibility by 2 independent reviewers. All disagreements were resolved by consensus. After the decision was made to include a paper, information was extracted on the first author, year of publication, research location, sample size, language, type of study, number and age of positive cases, diagnostic tests, and type of antibody using a data extraction form.

### Quality assessment

The Newcastle-Ottawa Scale [18] was used to assess the quality of the included studies. This tool assigns to a case-control study a maximum of 9 points in 3 different categories, including selection (0-4 points), comparability (0-2 points), and exposure (0-3 points). For cross-sectional and cohort studies, a maximum of 7 points can be awarded, divided among the 3 categories of selection (0-3 points), comparability (0-2 points), and outcome (0-2 points). On this basis, case-control and cohort studies with scores of 7-9, 4-6, and ≤3 and cross-sectional studies with scores of 6-7, 3-5, and 1-2 were assessed as good, fair, and poor, respectively.

### Meta-analysis

In this study, forest plots were used to estimate the pooled effect size and effect of each study with confidence intervals (CIs) to provide a visual summary of the data. In addition, common approaches, including the Cochran Q test and I^2^ statistic, were used to evaluate heterogeneity among the studies, with I^2^ values of <25%, 25-50%, and >50% considered to indicate low, moderate, and high heterogeneity, respectively. The assessment of heterogeneity and a fixed-effects model (Mantel-Haenszel) were used to compute the overall effect [19,20].

In addition, the possibility of publication bias was evaluated using the Egger regression test (a quantitative method). The meta-analysis was conducted using StatsDirect statistical software (https://www.statsdirect.com/). A p-value<0.05 was considered to indicate statistical significance. Additionally, subgroup analyses were performed for diagnostic methods and sample size.

The study protocol (CRD42017069384) was registered on PROSPERO, an international prospective register of systematic reviews [21].

### Ethics statement

This article is an approved plan (No. 73) from the Deputy of Research, Mazandaran University of Medical Sciences, Sari, Iran.

## RESULTS

### Literature search

**Figure 1.**
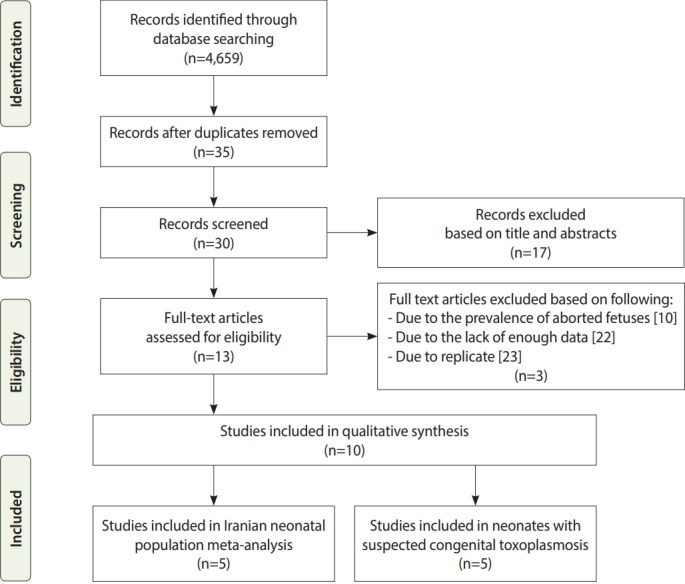
The PRISMA (Preferred Reporting Items for Systematic Reviews and Meta-Analyses) flow diagram of the search strategy, study selection, and data management procedure of *Toxoplasma gondii* infection in neonates in Iran.

The literature search resulted in the retrieval of 4,659 studies, 11 records of which were eligible for inclusion in the systematic review and 10 articles were entered into the meta-analysis. Out of the 10 articles, 5 were included in the meta-analysis of newborns, and 5 other studies were included in the meta-analysis of infants with suspected CT (Figure 1).

Among the 11 included studies, 7 and 4 articles were written in the English and Persian languages, respectively. Table 1 presents the results of the literature search and the characteristics of each study, including first author, year of publication, research setting, sample size, the number of the positive cases of serum IgG and IgM or PCR in neonates, method, and design.

Three different serological and molecular diagnostic tests—immunofluorescence assays (IFA), enzyme-linked immunosorbent assays (ELISA), and PCR—were used in the included studies to evaluate neonatal toxoplasmosis in Iran. Serological techniques were implemented in 10 studies for the diagnosis of *T. gondii*. PCR was used along with serological methods in 4 studies for the diagnosis of *T. gondii* infections in blood and cord blood samples. In addition, 1 study used only PCR to detect *T. gondii* in amniotic fluid. The publications included in this systematic review were conducted in 6 cities of Iran, namely Tehran (n=6), Isfahan (n=1), Gorgan (n=1), Rafsanjan (n=1), Kashan (n=1), and Arak (n=1).

### Congenital toxoplasmosis in Iranian neonates

Little variation was found in the frequency of anti-*T. gondii* IgM antibody positivity in the included studies among newborns (χ^2^(4)=3.37, p=0.49; I²=0.0%) and in infants with suspected CT (χ^2^(4)=6.65, p=0.15; I²=39.9%) in Iran. A total of 2,230 neonates were evaluated for toxoplasmosis through January 1, 2018, in different regions of Iran. In the studies that used only serological methods to diagnose the infection, the number of IgM-positive cases was utilized as the criterion to assess the incidence of CT. In addition, in studies where a molecular method was used for diagnosis, the number of PCR-positive cases was used to determine the incidence of CT.

**Figure 2.**
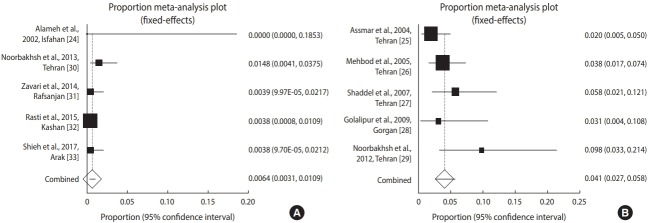
Forest plot diagram of the studies showing incidence rates of *Toxoplasma* infection in (A) neonates and (B) neonates with suspected congenital toxoplasmosis.

As shown in Figure 2, the incidence of toxoplasmosis in Iranian neonates (n=1,601) in these studies varied from 0.00% to 1.48%, with an overall incidence of 0.64% (95% CI, 0.31 to 1.09).

However, based on the meta-analysis using a fixed-effects model, the total incidence of this disease in neonates with suspected CT (n=629) was 4.10% (95% CI, 2.68 to 5.77), with a range from 2.0% to 9.8% across different studies (Figure 2). In this regard, the weighted incidence of CT was higher in infants with a suspected congenital infection than in neonates overall. Figure 2 presents the forest plot diagrams of this review.

### Subgroup analyses

The pooled incidence rates of *T. gondii* infection in Iranian neonates according to the diagnostic methods (ELISA, PCR, and IFA) were determined to be 0.7% (95% CI, 0.3 to 1.2), 0.7% (95% CI, 0.3 to 1.3), and 0.0%, respectively. In addition, the pooled incidence rates of CT in Iranian neonates in studies with sample sizes of <265 and ≥265 were 0.5% (95% CI, 0.1 to 1.4) and 0.6% (95% CI, 0.2 to 1.2), respectively.

The pooled incidence rates of *T. gondii* infection in neonates with suspected CT according to the diagnostic methods (ELISA, PCR, and IFA) were determined to be 3.6% (95% CI, 0.8 to 8.1), 3.7% (95% CI, 0.9 to 8.3), and 4.4% (95% CI, 2.4 to 6.9), respectively. Furthermore, the pooled incidence rates of *T. gondii* infection in neonates with suspected CT in studies with sample sizes of <150 and ≥150 were 6.3% (95% CI, 3.4 to 9.8) and 3.1% (95% CI, 0.1 to 4.9), respectively. The subgroup analyses revealed no statistically significant difference in the overall incidence of *T. gondii* in Iranian neonates based on the diagnostic method (χ^2^=0.03, p=0.854) or sample size (χ^2^=0.49, p=0.479). In the same vein, no statistically significant differences were found in the overall incidence of *T. gondii* among neonates with suspected CT according to the diagnostic method (χ^2^=0.61, p=0.736) or sample size (χ^2^=3.38, p=0.065).

### Risk of bias assessment

The articles included in this meta-analysis showed an acceptable quality. Table 2 presents the quality of the included studies, with a higher score indicating better quality.

**Figure 3.**
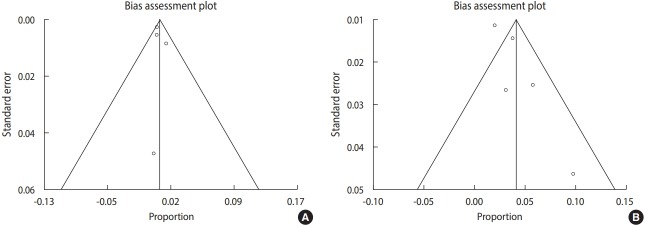
Bias assessment plot from Egger in the meta-analysis in (A) neonates, (B) neonates with suspected congenital toxoplasmosis.

In Figure 3, the Egger regression test and funnel plot revealed that publication bias exerted no significant influence on the overall incidence of *T. gondii* infection in the overall Iranian neonatal population and in suspected infants (p=0.48 and p=0.06, respectively).

## DISCUSSION

Toxoplasmosis is one of the most common human infections worldwide that can be transmitted from a mother to a newborn [13]. The reactivation of infection in immunocompromised women and the incidence of primary *Toxoplasma* infection during pregnancy can result in CT [34,35]. CT remains a major public health problem in Iran and other countries [36]. The purpose of this systematic review and meta-analysis was to evaluate the incidence of toxoplasmosis among neonates in Iran.

To the best of our knowledge, only 1 review article has been published before on the prevalence of toxoplasmosis among infants in Iran [37]. However, the present systematic review and meta-analysis focused on the incidence of *T. gondii* infection in infants suspected to have CT and in the overall neonatal population in Iran separately. This study involved the review of 11 articles found as a result of searching 7 databases. Our meta-analysis was carried out using 10 of the 11 reviewed articles. A total of 2,230 neonates, including 1,601 healthy Iranian neonates and 629 neonates with suspected CT were investigated for CT, and 444 were IgG-positive. In a study by Shaddel et al. [27], the number of positive cases was calculated based on ELISA and IFA findings. However, since the majority of the included studies used ELISA, the IgG-positive cases based on ELISA from the study of Shaddel et al. [27] were included in the meta-analysis.

Once a pregnant woman is confirmed to have toxoplasmosis, the possibility of a fetal infection should be investigated [13]. Serological tests are the usual diagnostic techniques for CT. However, these methods sometimes cannot detect anti-*Toxoplasma* antibodies during the acute phase of the disease [38]. The serological diagnosis of CT may be difficult because maternal IgG can cross the placenta and remain in the circulation of the newborn for several months. However, since the IgM antibody cannot cross the placenta, the presence of IgM at birth or several months after birth is suggestive of CT [27].

In the presence of relevant clinical findings in neonates, even with negative results for both IgM and IgG, toxoplasmosis is considered to be possible, and treatment is recommended [39]. The prenatal diagnosis of CT can be made by the PCR analysis of the amniotic fluid, which is safer and more sensitive than analysis of a fetal blood sample [13,39]. Furthermore, PCR can be carried out on a placental sample and umbilical cord blood to diagnose CT at birth. However, PCR analysis of the placenta renders more precise results in this regard [33]. The detection of *Toxoplasma* DNA in the placental tissue is not exclusively related to abortion, because the parasite may have only entered the placenta, but not the fetus [10].

The studies included in our meta-analysis were mostly conducted in the northern and central regions of Iran. In addition, 1 study was performed in the south of Iran (Rafsanjan). However, data gaps were identified for the west, east, and other parts of northern and southern Iran where no data were available. The weighted overall incidence rates of toxoplasmosis in the overall Iranian neonatal population and neonates with suspected CT were estimated to be 0.64% (95% CI, 0.31 to 1.09) and 4.10% (95% CI, 2.68 to 5.77), respectively, which exceed the global incidence of CT (estimated at 0.15% of live births) [40].

Mizani et al. [41] reported a high prevalence of *Toxoplasma* infection in Iranian pregnant women (43%), as well as girls and women of childbearing age (33%). In their study, 57% of pregnant women and 67% of girls and women of childbearing age were seronegative; therefore, they were susceptible to infection and needed to be monitored in this regard. Iran does not have a national program to investigate prenatal *T. gondii* infections at health institutions.

The analysis of the reviewed studies revealed that of the 6 cities in Iran for which data existed, Tehran and Isfahan had the highest and lowest incidence rates of *T. gondii* infection, respectively. Variation in the sensitivity and specificity of ELISA kits and the use of different cut-off values, age groups, geographic regions, and ethnic groups are among the factors affecting the reported incidence of infection [42-45].

Torgerson & Mastroiacovo [40] reported that the global annual CT incidence was 0.15% (of all live births). They also reported a high disease burden in South America, as well as some Middle Eastern and low-income countries. Individuals with negative results for both anti-*Toxoplasma* IgG and IgM have no serological evidence of prior exposure to *T. gondii*, and consequently are at a high risk of CT. Therefore, such patients require serial testing during gestation. Anti-*Toxoplasma* IgG-positive women should be tested for the IgM antibody, and in case of positivity, they should receive confirmatory tests.

Gestational age is very important in women with IgG-positive and IgM-negative antibodies. In IgG-negative and IgM-positive cases, a serological test should be repeated after 3 weeks. In this group, 2 outcomes can occur: the discrepancy in the antibody titer may persist (IgG-negative and IgM-positive) or the IgG antibody may change to positive (IgG-positive and IgM-positive) [5,41].

The management of the first group is the same as that of IgG- and IgM-negative patients, and the second group may undergo seroconversion. This group has a high risk of *T. gondii* transmission to the fetus; therefore, they should receive treatment, PCR analysis of amniotic fluid, and ultrasound examinations to limit the potential damage to the newborn [41].

The results of this study showed that neonates with suspected CT (4.10%) had a higher incidence of *T. gondii* infection than the overall neonatal population (0.64%) in Iran. CT may result in neurological and clinical manifestations. Some of the neurological manifestations of this infection include diffuse cerebral calcifications, convulsions, hydrocephaly, and microcephaly. In addition, the clinical manifestations of toxoplasmosis in newborns include hepatosplenomegaly, jaundice, lymphadenomegaly, thrombocytopenia, and anemia [46,47]. The positive period may be quite short in newborns with anti-*Toxoplasma* IgM; in addition, newborns with CT can have negative anti-*Toxoplasma* IgM at birth [48]. In a study conducted by Alameh & Tavangar [24], 7 newborns with anti-*Toxoplasma* IgG did not have the IgM antibodies; however, only 1 neonate had microcephaly and hypotony. The detection of *Toxoplasma*-specific IgA is more sensitive than IgM antibody detection in congenitally infected neonates. Infants suspected to have CT should be tested with both IgM-capture and IgA-capture ELISA [46] in order to limit the potential damage in this group.

There are numerous risk factors for toxoplasmosis in pregnant women and infants, including keeping cats (the most important risk factor), contact with infected livestock (e.g., cattle, sheep, and goats), goat milk consumption, place of residence (i.e., rural areas), gestation period, raw meat consumption, and vegetable/fruit consumption. However, most of the risk factors were not elaborated upon in the reviewed studies. Therefore, we eschewed the meta-analysis of these risk factors, which is among the limitations of this study.

Our review indicated that CT is not monitored in the healthcare programs implemented in Iran. Therefore, more studies are required for monitoring *Toxoplasma* infections among newborns in the future. Moreover, monitoring should continue in newborns with CT who are negative for anti-*Toxoplasma* IgM. One of the limitations of the present systematic review and meta-analysis is the non-evaluation of the related risk factors in the majority of papers. In addition, the included studies did not have a uniform sample size. The reviewed studies used different methods for the prenatal and neonatal diagnosis of CT, leading to dissimilar results. Additionally, studies in this field were not conducted in all regions of the country. These limitations may have serious ramifications regarding the epidemiological aspects of toxoplasmosis among neonates in Iran. It is very important to utilize a particular detection method, instead of various assays with different sensitivities, specificities, and cut-off values, to obtain more precise results. In addition, many questions remain to be answered in future investigations.

## CONCLUSION

This is the first systematic review and meta-analysis to provide a summary of the available data on the incidence of *T. gondii* infection in the Iranian neonatal population. Based on the included studies, the incidence of *T. gondii* is high among Iranian neonates. The high incidence of *Toxoplasma* infection in this population shows that CT is a public health problem that has been ignored by the Ministry of Health. Therefore, further studies are needed to estimate the incidence of CT in newborns and to determine its risk factors in Iran through the implementation of screening and detection programs using standardized methods.

## Figures and Tables

**Table 1.** Characteristics of the included studies of *Toxoplasma gondii* infection in neonates in Iran

Study	City	Neonates (n)	IgG (n)	IgM (n)	PCR (n)	Method	Design
Alameh et al., 2002 [24]	Isfahan	18	7	0	-	IFA	Cross-sectional
Gharavi, 2003 [22]	Tehran	18	18	8	-	IFA, ISAGA	Cross-sectional
Assmar et al., 2004 [25]	Tehran	11	11	4	4	PCR	Cross-sectional
Mehbod et al., 2005 [26]	Tehran	210	79	8	-	ELISA, IFA	Cross-sectional
Shaddel et al., 2007 [27]	Tehran	104	ELISA: 38, IFA: 60	ELISA: 6, IFA: 5, total: 7	6	ELISA, IFA, PCR	Cross-sectional
Golalipour et al., 2009 [28]	Gorgan	64	23	2	-	ELISA	Cross-sectional
Noorbakhsh et al., 2012 [29]	Tehran	50	9	5	-	ELISA	Case-control
Noorbakhsh et al., 2013 [30]	Tehran	270	119	4	0	ELISA, PCR	Cohort
Zavari et al., 2015 [31]	Rafsanjan	254	83	1	-	ELISA	Cross-sectional
Rasti et al., 2015 [32]	Kashan	9	9	0	3	ELISA, PCR	Cohort
Shieh et al., 2017 [33]	Arak	261	37	3	1	ELISA, PCR	Cross-sectional

IgG, immunoglobulin G; IgM, immunoglobulin M; PCR, polymerase chain reaction; IFA, immunofluorescence assay; ISAGA, immunosorbent agglutination assay; ELISA, enzyme-linked immunosorbent assay.

**Table 2.** Quality assessment of the included studies based on the Newcastle-Ottawa Scale^1^

Study	Selection (3 points)	Comparability (2 points)	Outcome (3 points)	Total score
Alameh et al., 2002 [24]	3	1	2	6
Gharavi, 2003 [22]	3	1	1	5
Assmar et al., 2004 [25]	2	1	1	4
Mehbod et al., 2005 [26]	3	1	1	5
Shaddel et al., 2007 [27]	2	0	1	3
Golalipour et al., 2009 [28]	2	1	1	4
Noorbakhsh et al., 2012 [29]	3	2	1	6
Noorbakhsh et al., 2013 [30]	3	2	2	7
Zavari et al., 2015 [31]	3	2	2	7
Rasti et al., 2015 [32]	2	2	2	6
Shieh et al., 2017 [33]	3	2	2	7

1 Good: 7-9; fair: 4-6; poor: ≤3 for case-control studies and good: 6-7; fair: 3-5; poor: 1-2 for cross-sectional studies.
